# Heavy metal pollution in Manzala Lake sediments, Egypt: sources, variability, and assessment

**DOI:** 10.1007/s10661-022-10081-0

**Published:** 2022-05-17

**Authors:** Mostafa Redwan, Engy Elhaddad

**Affiliations:** 1 Geology Dept, Faculty of Science, Sohag University, Sohag, 82524 Egypt; 2 National Institute of Oceanography and Fisheries, Cairo, Egypt

**Keywords:** Environmental pollution, Heavy metals, Manzala Lake, Egypt, Principal component analysis, Waste disposal

## Abstract

The environmental pollution of lake systems due to anthropogenic factors is of growing concern worldwide. Manzala Lake is the largest northern coastal-deltaic lakes of Egypt and has socioeconomic impacts. In this study, the concentrations and origins of seven heavy metals (HMs) and the organic content in the Manzala Lake sediments were explored during the winter and summer. The concentration of the HMs and the organic content were quantified using inductively coupled plasma and loss-on-ignition techniques. Pearson’s correlation coefficient (PCC) and principal component analysis (PCA) were applied to evaluate the sources of the metals in the sediments. The HMs and organic matter were enriched during the winter season. The average concentrations of the HMs in the sediments conformed to the following sequence: Fe (14.13) > Mn (0.8) > Cu (0.11) > Zn (0.11) > Ni (0.06) > Pb (0.5) > Cd (0.002) (mg/kg). Sediment quality protocols showed that Mn, Cd, Cu, and Ni pose a significant threat to the aquatic environment in Manzala Lake. The geoaccumulation index (*I*_geo_) values indicated pollution of the sediments with most metals, excluding Fe and Ni. The periodic mean *I*_geo_ pollution level followed the sequence Cd > Cu > Zn > Mn > Pb > Ni > Fe. The greatest pollution load index noted during the winter season was principally induced by Cd and Cu. The overall ecological risk index was moderate, with Cd being the most prominent HM. PCA combined with PCC showed that the HM enrichments in the southern (Bahr Al-Baqar Drain [S1], Bashteer [S3], Legan [S5], and Al-Ginka [S8]) and the extreme northeastern (El-Qapouti [S6]) parts of Manzala Lake sediments were mainly due to the discharge from different drains (industrial, agricultural, and municipal wastes) and the industrial region in Port Said, respectively. The lower HM concentrations from the extreme northern parts (Al-Boghaz [S2], Al-Temsah [S4], Al-Hamra [S7], and Al-Kowar [S9]) were due to their isolation from urban areas compared with the other localities. Extensive waste disposal was responsible for the HM pollution in the Manzala Lake sediments. Advanced treatment technologies and monitoring of the pollution in the water and sediments of Manzala Lake are required to decrease the accumulation of the heavy metals.

## Introduction

Toxic heavy metals (HMs) are common environmental pollutants given their environmental tenacity, high toxicity, and bioaccumulation capability, causing notable threats to both aquatic life and water quality. The pollution of lake-bottom sediments by HMs is considered one of the greatest issues in environmental science because of their possible biotic toxicity, environmental endurance, and accumulation (Varol, 2011; Jiang et al., 2012; Ergül et al., 2013; Xu et al., 2017). HMs primarily penetrate the aquatic environments because of different factors (e.g., industrial, agricultural, combustion, and smelting factors, sewage effluents, and road vehicles activities) (Rajeshkumar et al., 2018). Different negative health impacts, such as cancer, intellectual and developmental disabilities, impaired intelligence, kidney damage, and stillbirth, are linked to HM exposure (Alomary & Belhadj, 2007; Rinklebe et al., 2019).

HM adsorption on sediments in the water column can lower the pollution level of water. Great quantities of organic matter in sediments can take up HMs from the water. Therefore, sediments can trap HMs, elevating their HM content (Hahn et al., 2018). Sediment pollution can be catastrophic as it is the principal habitat and food source for benthic biota. Gibbs (1973) concluded that more than 30% of the entire metal weight in lakes settle in bottom sediments and that HM concentrations in sediments are higher than those above the water column by up to five times. However, variations in environmental conditions such as conductivity, temperature, pH, organic complexing agents, grain sizes, redox conditions, and pollutants can liberate contaminant loads into the water and cause secondary pollution (Eggleton & Thomas, 2004; Fu et al., 2014; Zhang et al., 2016; Malvandi, 2017). The bioaccumulation of HMs is detrimental to terrestrial organisms and human beings (Kaushik et al., 2009).

**Fig. 1:**
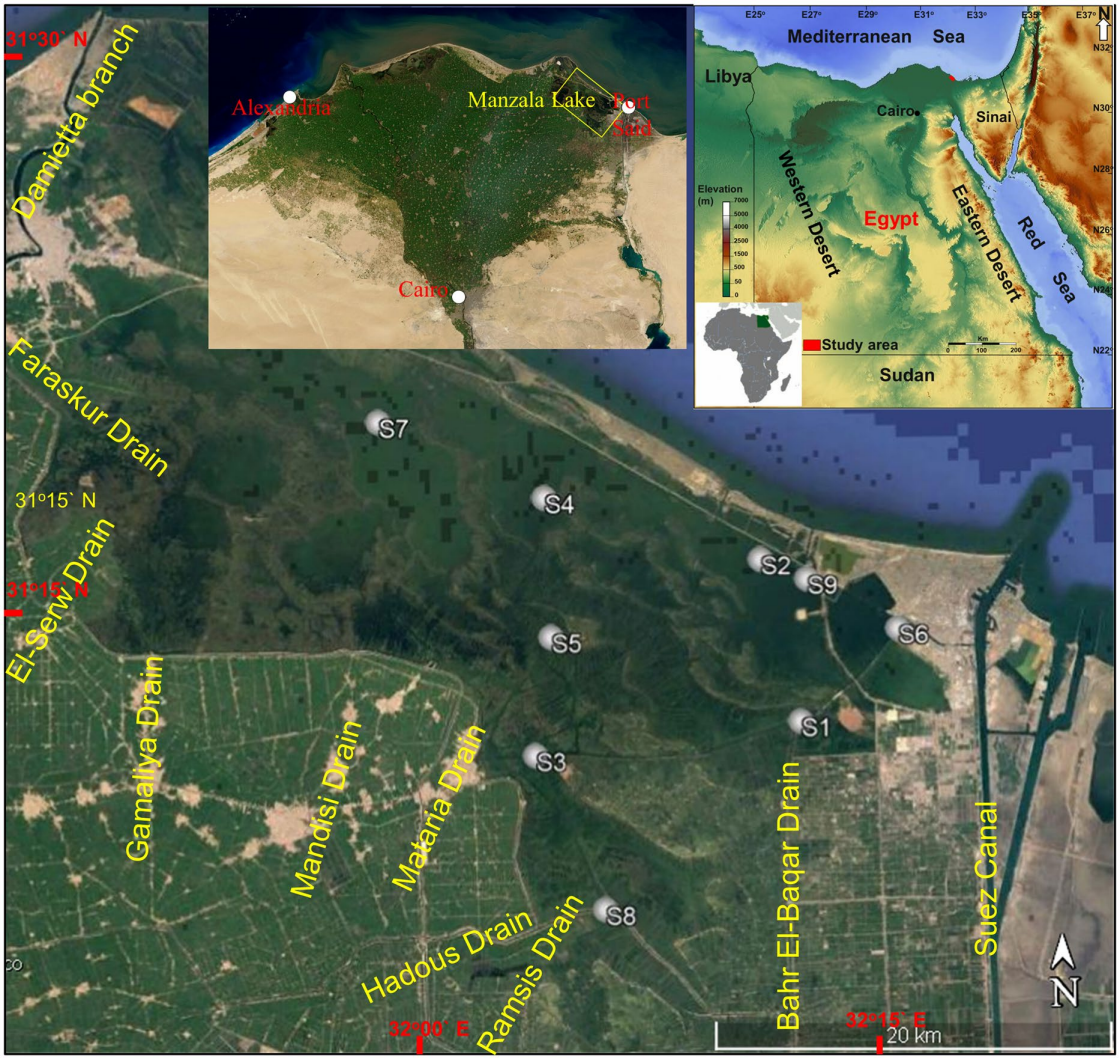
Study area and sampling locations are shown with grey circles in Manzala Lake, Egypt

The Northern Delta lakes of Egypt on the Mediterranean coast comprise the Edku, Burullus, Manzala, and Mariut, covering around 6% of the non-desert area of Egypt. They are considered critical natural reserves for fish production (tilapia species) in the country. Lake Manzala is the greatest of the Delta lakes of the Nile River, bounded by the latitudes of 31°10′ and 31°40′N and longitudes of 31°50′ and 32°25′E. It is situated on the northeastern corner of the Delta, about 170 km from Cairo City and about 15 km west of Port Said City (Fig. 1). The lake is bounded by the Mediterranean Sea from the north, the Suez Canal from the east, Port Said to the northeast, Dakahlia from the southwest, Sharkia from the south, and Damietta from the west. The lake surface area was reduced from 1709 km^2^ in 1907 to 1470 km^2^ in 1949, 1260 km^2^ in 1960, and 895km^2^ in 1979 (Mageed, 2007) because of ongoing land reclamation projects and the creation of fish farms, especially in the southern areas. The total water body loss in Manzala Lake was around 355km^2^ between 2003 and 2012, with the decrease expected to be around 84.67% in 2030 (Negm & Hossen, 2016). Lake Manzala is brackish, shallow, and elongated, with a mean depth of 1.2–1.5 m and a length of 60 km (El-Kholy et al., 2012). Mediterranean seawater invades the lake from the opening of Boughaz El-Gamil to the west of Port Said and the Sheikh Ali passage 25 km east of the Damietta Governorate. Because of the low freshwater inputs to the lake, HMs accumulate over time in the system.

Manzala Lake provides over 30% of the commercial fish in Egypt. However, Manzala Lake showed high pollution rates in the last six decades due to different contaminant sources (agriculture, sewage, and industrial wastewater), especially from the southern and western borders (Wahaab & Badawy, 2004). These contaminants greatly polluted the lake, devastating the commercial production of fish and natural resources (Zahran et al., 2015).

The lake gains drainage water from six major heavily polluted drains, Bahr El-Baqar, Hadous, El-Serw, Ramsis, Mataria, and Faraskur, with a total discharge of about 4000 million m^3^/year (Hegazy et al., 2016). The Bahr El-Baqar drain and Hadous present about 75% of the total waste input into Manzala Lake, with pollution sources from treated and raw industrial wastewater, sewage, and drainage of some agricultural areas (Stahl et al., 2009; Mohamedein et al., 2018; Elsaeed et al., 2020). The Hadous, El-Serw, Ramsis, and Faraskur drains discharge agricultural water to the lake. The Mataria drain discharges sewage wastes (Shakweer, 2005).

Accordingly, examining the deterioration of lake-bottom sediments and appraising the ecological risks caused by HMs are crucial in preventing long term impacts on aquatic ecosystems and human health. The main objectives of this study were (1) to appraise the concentrations and sources of the HMs in the bottom sediments of Manzala Lake and (2) to evaluate the ecological risks posed by these polluted sediments and possible environmental management strategies.

## Materials and methods

Big regions northwest of Manzala Lake are used as fish farms. Meanwhile, the southern areas are used for agriculture. The sediments of the lake comprise sand, silt, clay, or shell accumulations of lamellibranch. The lake is exposed to gradual flows of contaminants from many drains.

### Sampling methods

Surficial sediment samples (topmost 5 cm of the bottom sediments) were gathered from Manzalla Lake in the winter and summer of 2020 using an Ekman dredge sampler from nine stations: S1 (Bahr Al-Baqar drain), S2 (Al-Boghaz), S3 (Bashteer), S4 (Al-Temsah), S5 (Legan), S6 (El-Qapouti), S7 (Al-Hamra), S8 (Al-Ginka), and S9 (Al-Kowar) (Fig. 1). GPS Garmin Dakota 10 was used to record the coordinates of each sampling station. Samples transported to the laboratory in polyethylene bags. Then the samples were air-dried at room temperature, sieved to attain <63-µm fractions, homogenized, ground in an agate grinder, and stored at − 15 °C until analysis.

### Analysis of sediment samples

The sediments were dried in an oven at 105 °C for 1 day. The organic matter content was evaluated using the loss-on-ignition approach through weight difference (Kristensen & Andersen, 1987). Approximately 0.5 g of pulverized sediment was digested using a mixture of 4.0 mL of HNO_3_, 4.0 mL of HClO_4_, and 15.0 mL of HF in a Teflon beaker. The reagents and acids used were of analytical grade, following the Environmental Quality and Quality Control Standard for soil contamination (APHA, 1989). Standard reference sediment samples were applied during the measurements (CANMET SRSD-1 and 3; Lynch, 1990). Reagent blanks were included for in-house laboratory quality assurance and control. All samples were prepared and analyzed in duplicates in parallel with an error of less than 5% to ensure the accuracy of the results. Seven HMs (Fe, Mn, Cd, Cu, Ni, Pb, and Zn) were estimated using inductively coupled plasma–mass spectrometry (ICP-MS, Model: Elan 9000, Perkin Elmer, Waltham, MA, USA) with detection limits of < 0.0001 mg/g.

### Heavy metal assessment in sediment

#### Geoaccumulation index (I_geo_)

*I*_geo_, formulated by Müller (1969) to measure the enrichment of HMs in sediments by comparing the element levels to their background preindustrial concentrations (Eq. 1), is applied herein.

1 $${I}_{\mathrm{geo}}={\mathrm{log}}_{2}\left(\frac{{C}_{m}}{{1.5 C}_{n}}\right)$$

where *C*_*m*_ is the toxic element content, *C*_*n*_ is the mean natural background level for the element in a reference shale (Turekian & Wedepohl, 1961), and 1.5 is a correction factor due to the lithogenic and weathering impact (Taylor, 1964). The sediments can be grouped into seven classes on the basis of their *I*_geo_ values: ≤ 0, unpolluted; ≤ 1, unpolluted to moderately polluted; ≤ 2, moderately polluted; ≤ 3, moderately to highly polluted; ≤ 4, highly polluted; ≤ 5, highly to very highly polluted; > 5, very highly polluted (Chen et al., 2007; Müller, 1969).

#### Tomlinson pollution load index (PLI)

The contamination factor (*CF*) estimates metal enrichment in sediments in relation to a background value (Eq. 2). The *PLI* assesses the total pollution grade and sediment toxicity in the study area (Eq. 3) (Tomlinson et al., 1980):

2 $$CF=\frac{{C}_{m}}{{C}_{n}}$$

3 $$PLI =\sqrt[n]{{CF}_{1}{ \times CF}_{2} \times {\dots \dots .\times CF}_{n}}$$

where *C*_*m*_ and *C*_*n*_ are as defined earlier, and *n* is a metal’s number. A *PLI* of < 1 implies no existing metal pollution, *PLI* = 1 indicates minimum pollution, and *PLI* > 1 means quality degradation of the location (Tomlinson et al., 1980).

#### Potential ecological risk index (RI)

*RI*, recommended by Håkanson (1980), was utilized to quantify the degree of ecological risk (*Er*) level (Eq. 4) of HMs in sediments. *RI* appraises the environmental and ecological toxicity of numerous HMs (Eq. 5). *Er* (Eq. 4) is estimated from *CF* (Eq. 2).

4 $$Er\hspace{0.17em}=\hspace{0.17em}Tr\hspace{0.17em}\times \hspace{0.17em}CF$$

5 $$RI=\sum {Er}_{i}$$

where *E*_*r*_ and *T*_*r*_ are the risk factor and the toxic response factor of an HM *i* (the *T*_*r*_ values for Cd, As, Ni, Pb, Cu, Cr, and Zn were 30, 10, 5, 5, 5, 2, and 1, respectively) for a single metal *i* (Cheng & Yap, 2015; Håkanson, 1980). Five categories were identified according to the potential ecological risk (Håkanson, 1980): *Er* < 40, low level; 40 ≤ *Er* < 80, moderate level; 80 ≤ *Er* < 160, considerable level; 160 ≤ *Er* < 320, high level; and *Er* ≥ 320, very high level. Four categories were identified according to the *RI*: *RI* < 150, low ecological risk; 150 ≤ *RI* < 300, moderate ecological risk; 300 ≤ *RI* < 600, considerably high ecological risk; and *RI* ≥ 600, very high ecological risk.

### Statistical analysis

Pearson’s correlation coefficient (PCC) and principal component analysis (PCA) were utilized using the statistiXL software (www.statistixl.com/) (Ma et al., 2015) to ascertain the sources of the HMs. PCA was utilized to define the data in a simple understandable form and to classify the different processes affecting the composition of the sediments. Data normalization and standardization were applied to let the variables have the same weight during analyses. Before PCA analysis, the Kaiser–Meyer–Olkin test and Bartlett’s test were executed to assess the adequacy of the metals data for factor analysis and the structure of variability between the metals and suitability for PCA (Sharma, 1996). Principal components with eigenvalues > 1 were retained for interpretation. A level of probability of 0.05 or less was considered significant (Hair et al., 1998). Factor loading computed the propinquity degree between each variable and factor. The variables with the largest absolute values point to a greater kinship among the particular factors and variables (Armstrong et al., 2013).

## Results and discussion

### HM pollution assessments and variations

The prevailing type of sediment in Manzala Lake was silt and clay intercalation. The concentrations of the seven HMs and organic matter in the bottom sediments from the study area during the winter and summer seasons and from the reference lakes are summarized in Table 1. The last destinations for natural and anthropogenic constituents gained from the surrounding environments were the lake-bottom sediments. The quality of sediments determined whether the water column was polluted or not by HMs and different other organic contaminants.

**Table 1:** HM concentration and organic content (%) values in Manzala Lake sediments in winter and summer (mg/g)

**mg/g**	**Fe**	**Mn**	**Cd**	**Cu**	**Ni**	**Pb**	**Zn**	**OM (%)**
**Winter**								
** Mean**	18.13	1.03	0.0021	0.13	0.06	0.02	0.12	16.33
** Min**	9.76	0.46	0.0010	0.07	0.04	0.01	0.08	7.45
** Max**	30.45	1.88	0.0040	0.26	0.08	0.04	0.23	26.34
**Summer**								
** Mean**	10.12	0.57	0.0016	0.10	0.05	0.01	0.10	10.34
** Min**	3.12	0.25	0.0010	0.06	0.03	0.01	0.06	4.7
** Max**	19.67	0.89	0.0030	0.21	0.07	0.03	0.19	18.9
**Periodic mean values**	14.13	0.80	0.0018	0.11	0.06	0.02	0.11	13.33
**Mariout Lake** (EL-Bady, 2020)	19.34	0.585	0.0007	0.091	0.04	0.059	0.139	—
**Edku Lake** (Waheshi et al., 2017)	38.82	1.923	—	0.072	0.05	0.045	0.083	4.90
**Burullus Lake** (Melegy et al., 2019)	17.55	0.948	0.0002	0.03	0.04	0.03	0.05	6.48
**Average shale** (Turekian & Wedepohl, 1961)	47.2	0.85	0.0003	0.045	0.068	0.02	0.095	—
**ERL** (Long & Morgan, 1990)	—	—	0.0012	0.03	0.021	0.05	0.15	—
**PEL** (MacDonald et al., 2000)	—	—	0.0042	0.108	n.a	0.112	0.271	—

The highest organic matter content was recognized at site S1 (26.34%) at the Bahr El-Baqar drain (the southern areas of the lake) during the winter season due to the direct discharge of treated and industrial (petroleum refining and associated chemical by-products; Barakat, 2004), sewage, and agricultural wastes and/or the spread of fisheries (El-Badry & Khalifa, 2017; Elsaeed et al., 2020; Stahl et al., 2009). The lowest values were determined at site S9 (4.7%) during the summer season in the extreme northeastern parts of the lake, which is far from immediate pollution (Table 1).

Bek et al. (2018) recorded that the dissolved oxygen concentrations decreased from their highest values during the winter to their lowest values during summer in the southern sector of the lake because of the high amount of wastewater discharges in this region and the high biological oxygen demand (BOD) associated with these. The high organic content in winter can be attributed to the presence of calcareous algae (Rifaat, 2005). The HM concentrations ranged from 0.0010 to 30.45 mg/g. The Holocene sediments (< 2 μm) of the Nile Delta were enriched in Fe, Mg, Ni, Cu, and Zn relative to the continental crust from basic rock provenance (Siegel et al., 1995). In the study area, Fe showed elevated concentrations during the winter compared with any other HM, followed by Mn, Cu, and Zn. Meanwhile, Cd had the lowest concentration values. The distribution pattern of Fe was mostly assigned to the influence of agricultural activities and domestic wastes discharged into the lake (Elmorsi et al., 2019). The results in Table 1 show the mean levels of HMs in the investigated area. It shows the following order of the HMs according to their concentrations: Fe > Mn > Cu > Zn > Ni > Pb > Cd.

**Fig. 2:**
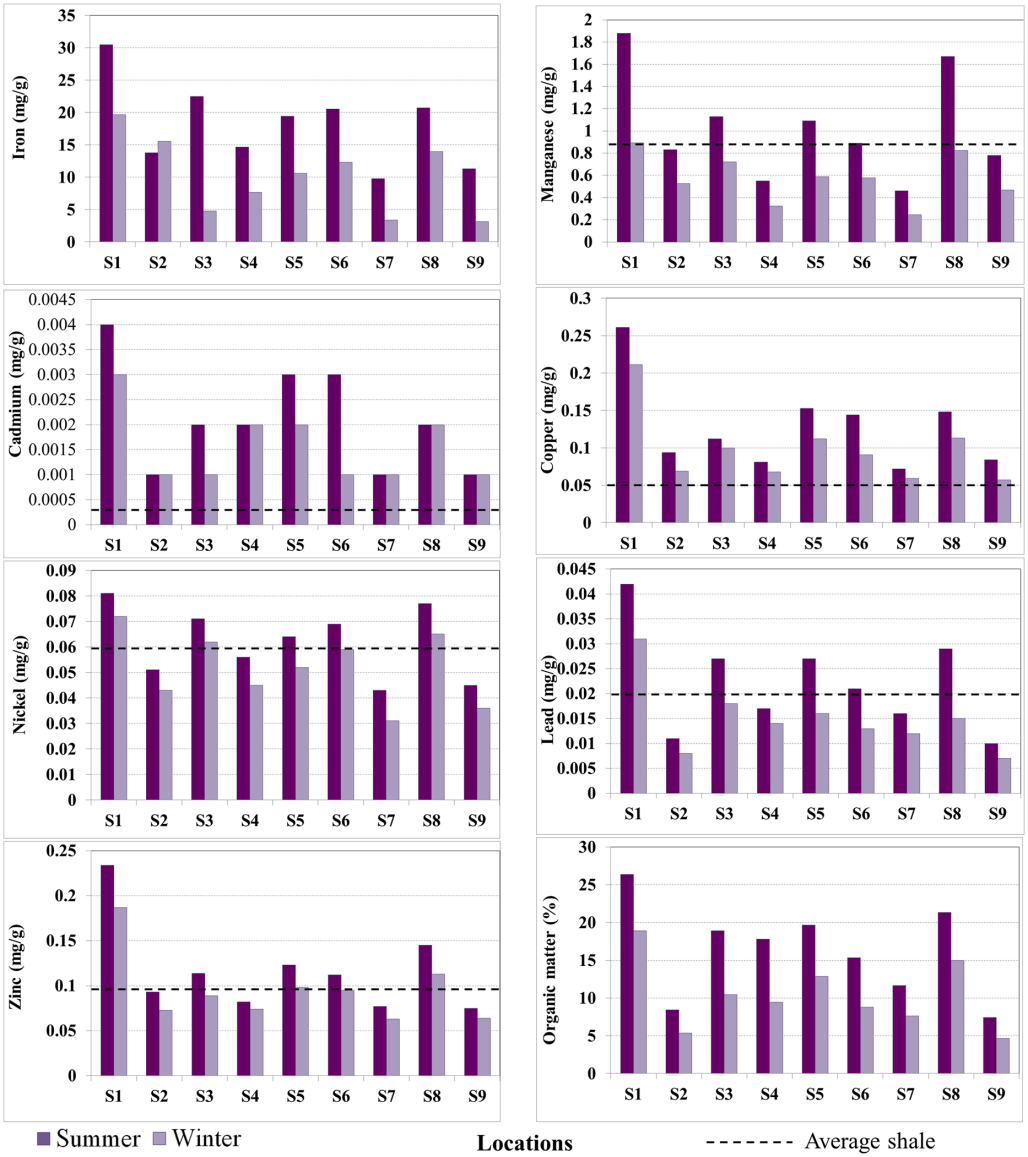
Total HM concentrations (mg/g) and organic matter (%) in sediments of Manzala Lake during winter and summer seasons. The dotted line represents average shale values (Turekian & Wedepohl, 1961)

Manzala Lake is situated in a low rainfall zone with an average annual rate of 78.4 mm and peak rainfall in the winter season. Large amounts of water discharged from the major drains in the lake occurred in summer, whereas the smallest rates were observed in winter. The discrepancy of the metal concentrations from month to month was affected by the drain discharge properties (Bek et al., 2018). The six major drains transferred about 5463 × 10^6^ m^3^ of water per year (Ayache et al., 2009) to the lake. The different high metal inputs were the main factors controlling the metal availability in the lake. Therefore, the HM concentrations were higher in winter (Table 1, Fig. 2). This is similar to that of the Nile River sediments and branches, where similar studies on HM variations in the Rosetta and Damietta branches (Redwan & Elhaddad, 2016, 2020) exhibited higher enrichment of HMs in winter, corresponding to the high mean water inflow from the Aswan High Dam (AHD) in summer (almost double) than in winter (Shamrukh & Abdel-Wahab, 2011).

The sediment quality regulations (effect range low [ERL] and probable effect level [PEL]), proposed by the US National Oceanic and Atmospheric Administration and Canadian Freshwater Sediment Guidelines (Long & Morgan, 1990; MacDonald et al., 2000; CCME, 2001) were chosen to analyze the HM-induced degradation and ecotoxicology of the ecosystem (Ma et al., 2015; Wei et al., 2019) in the bottom sediments of the Manzala Lake. The results are presented in Table 1. Impacts on living organisms scarcely happen if the metal value is lower than the ERL, whereas concentrations greater than the PEL indicate adverse effects. The Fe concentrations in the sediments were higher than those of any other metals. The average concentrations of Cu and Ni in winter and summer were higher than those of the average shale, ERL, and PEL. The Cd concentrations in winter and summer were greater than those of the average shale and ERL but lower than the PEL. The concentrations of Pb and Zn in winter and summer were higher than those of the average shale but lower than the ERL and PEL. This variable rise in the HM concentrations poses adverse higher toxicity to living organisms.

The maximum concentration of Fe (30.45 mg/g) was reported at the Bahr El-Baqar drain (S1) in winter, and the lowest value (3.12 mg/g) was registered et al.-Kowar (S9) in summer. S3, S5, and S8 were enriched with HMs because of the release of agricultural and sewage wastes from the Hadous and Mataria drains (Shakweer, 2005) and because of the presence of an industrial region in Port Said in the case of S6 (El-Qapouti). Similarly, Mn had its highest mean concentration value of 1.88 mg/g reported during winter at the Bahr El-Baqar drain (S1) attributed to industrial activities and its lowest value of 0.245 mg/g et al.-Hamra (S7) in the northern portion of the lake during summer. The high Mn concentrations were possibly due to iron and steel production, diesel combustion in boats (Beliles, 1979), agricultural drainage water rich in fertilizers, and high Mn residues from chicken farms for feeding fish (Hamed et al., 2013). According to Abdel-Moati and El-Sammak (1997), Fe and Mn exhibited the smallest metals that affected the pollution budgets in the Nile Delta lake sediments.

The highest mean concentrations of Cu, Zn, Ni, Pb, and Cd reported during winter were 0.261, 0.234, 0.081, 0.042, and 0.004 mg/g, respectively, at the Bahr El-Baqar drain (S1). In contrast, the lowest mean values of 0.057 (S9), 0.063 (S7), 0.31 (S7), 0.007 (S9), and 0.001 mg/g (S2–S3, S6–S7, and S9) were respectively reported in summer. Abdel-Baky et al. (1998) noted a positive association between organic matter and the HMs in the water.

Generally, Cd is more highly mobilized than most HMs in aquatic systems. Cd affects the ecosystem directly because of its noticeable toxicity as a result of its nondegradable bioavailability (Smolders et al., 1999). The use of pesticides and phosphate fertilizers from agricultural runoff increases its abundance (El Kammar et al., 1999; El-Badry, 2016; Venkatesha Raju et al., 2012).

Cd and Pb are largely related to phosphate fertilizers and some industries such as paint factories (El-Badry, 2016; Bahnasawy et al., 2011; Wei et al., 2019). In addition, dust holds a large amount of Pb from vehicle exhausts (Hardman et al., 1994) and disposal of gas factories nearby the lake (Hamed et al., 2013). Pb’s chemical activity depends chiefly upon the existence of organic matter at pH values greater than 4 (Kerndorff & Schnitzer, 1980).

The Cu and Ni values in sediments are influenced by agricultural and industrial discharges (Cempel & Nikel, 2006) and Pb enriched in industrial and sewage wastes (Singh et al., 2012) and many industrial applications such as house paint, plumbing pipes, and storage batteries (Junior et al., 2002; Thürmer et al., 2002). Domestic and industrial wastewaters bearing Cd, Cu, and Zn were deemed as great roots of these metals (Belabed et al., 2013). Also, the application of fungicides to citrus farms is the principal source of Cu in the Egyptian irrigation systems (Mason, 2002).

The HM enrichment in the southern (Bahr Al-Baqar drain [S1], Bashteer [S3], Legan [S5], and Al-Ginka [S8]) and extreme northeastern (El-Qapouti [S6]) parts of the Manzala Lake sediments was mainly due to point source pollution discharge from different drains (industrial, agricultural, and municipal wastes) and the industrial region in Port Said, respectively. The extreme northern parts (Al-Boghaz [S2], Al-Temsah [S4], Al-Hamra [S7], and Al-Kowar [S9]) were isolated from urban areas compared with the other localities, accordingly exhibiting lower HM concentrations.

The Mariout Lake was devastated by primary and raw sewage and raw industrial, domestic, and agricultural waste effluents (Elbehiry et al., 2018; Mohamedein et al., 2018). Meanwhile, Edku Lake gains input from diverse anthropogenic activities and Alexandria City wastewaters even though there is a minor intensity of industrial activities nearby (Mohamedein et al., 2018). Also, various sediment samples in Burullus Lake were devastated mainly by agricultural drainage water and little industrial and domestic waste discharges (El Baz, 2015).

The greater enrichments of HMs (e.g., Zn, Pb, Cu, and Ni; Table 1) in Mariout Lake than those of the other lakes were due to the various contaminant inputs to the lake. The Ni content in Burullus Lake may be attributed to mafic Quaternary Nile sediments (pyroxenes, amphiboles, and epidotes) (Mohamedein et al., 2018). The Fe and Mn contents in the sediments of Edku Lake are attributed to the large amount of organic matter and high volume of domestic discharge from boats and clay sediments enriched in Mn (Aston & Chester, 1973; Alaa et al., 2004) and their oxide and hydroxide associations (De Groot & Allersma, 1975).

The area facing the drainage in Manzala Lake is prevailed by fine-grained sediments and is rich in organic content, whereas the central parts are characterized by a mixture of sand, silt, clay, and low organic content, which may affect the content of HMs (Arnous & Hassan, 2015; Tasi et al., 2003).

### Heavy metal risk assessment in sediment

#### The geoaccumulation index

**Fig. 3:**
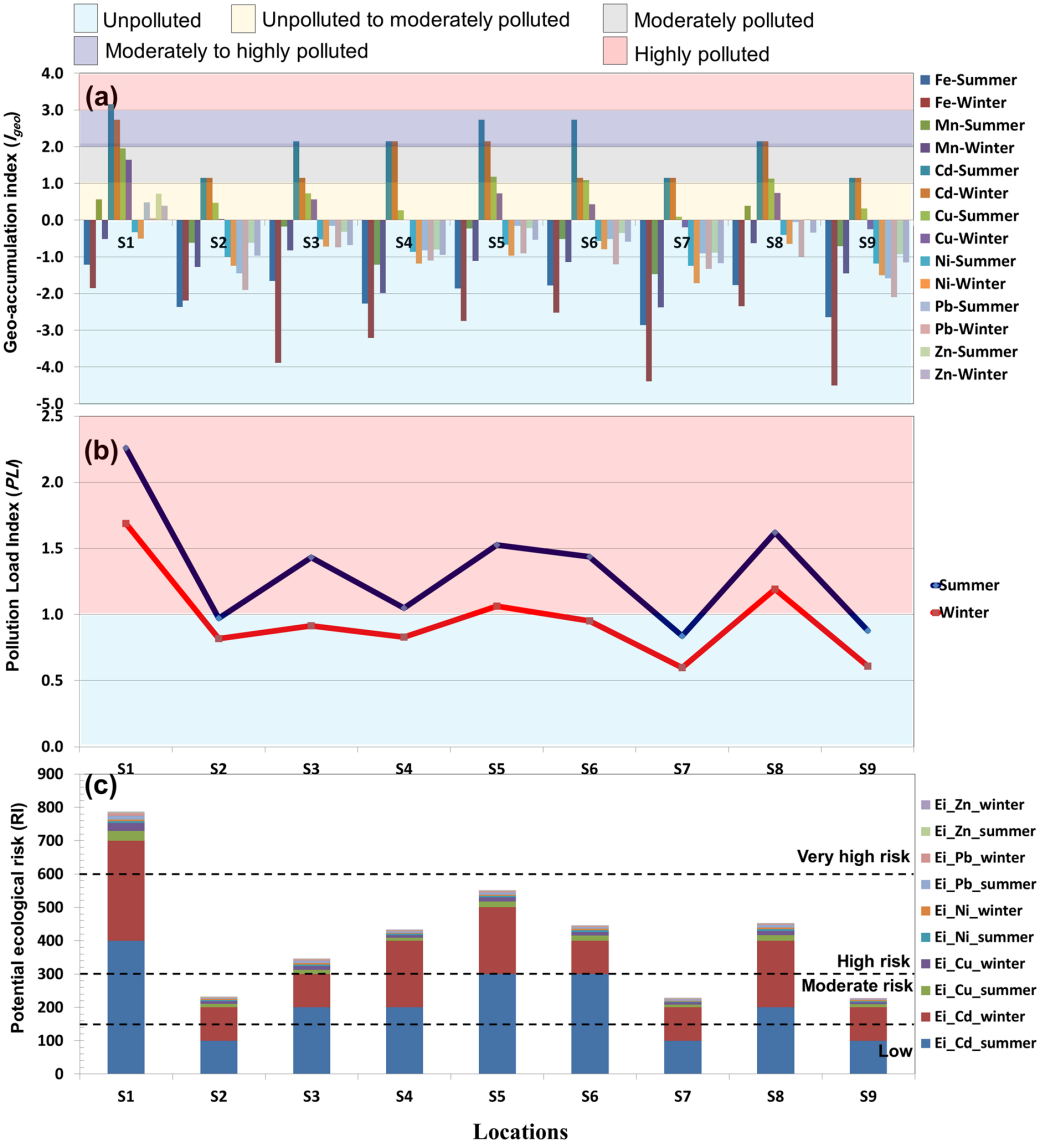
*I*_*geo*_ (**a**), *PLI* (**b**), and *RI* (**c**) of HMs in the sediments of Manzala Lake. Win, winter; Sum, summer

The calculated *I*_*geo*_ values of the HMs in the Manzala Lake sediments indicated pollution of the sediments with most metals other than Fe and Ni (Table 2, Fig. 3a). The Fe and Ni *I*_*geo*_ values in winter and summer indicate no pollution. In contrast, those of Mn, Pb, and Zn remained mostly in class 0 but became class 1 at site S1 during the winter and summer seasons and at site S8 during the winter season. The Cu *I*_*geo*_ values in winter and summer varied from class 0 (S7 and S9) to class 2 (site S1) and in winter to class 2 (S1, S5–S6, and S8). The Cd *I*_*geo*_ values ranged from class 2 in summer (sites S2–S3 and S6–S9) to class 3 (sites S1 and S4–S5) and in winter from class 2 (sites S2, S7, and S9) to class 3 (sites S3–S6 and S8) and class 4 (site S1), indicating the strong deterioration of the sediments with Cd notably during the winter times. The periodic average pollution level in the sediments followed the sequence Cd > Cu > Zn > Mn > Pb > Ni > Fe. Agricultural, municipal, and industrial wastes (Wei et al., 2019) in winter and summer in Manzala Lake were liable for the elevated sediment pollution. Therefore, Cu and Cd pollution and their sources should be paid attention to by the authorities and the local community because of their apparent risk to the aquatic system of Manzala Lake.

**Table 2:** *I*_*geo*_ and *PLI* values of different HMs in the sediments of Manzala Lake in winter and summer

***I***_**geo**_	**Fe**	**Mn**	**Cd**	**Cu**	**Ni**	**Pb**	**Zn**	***PLI***
**Winter**								
** Mean**	− 2.05	− 0.44	2.06	0.80	− 0.75	− 0.57	− 0.37	1.34
** Min**	− 2.86	− 1.47	1.15	0.09	− 1.25	− 1.58	− 0.93	0.84
** Max**	− 1.22	0.56	3.15	1.95	− 0.33	0.49	0.72	2.26
**Summer**								
** Mean**	− 3.07	− 1.26	1.66	0.41	− 1.03	− 1.14	− 0.67	0.96
** Min**	− 4.50	− 2.38	1.15	− 0.24	− 1.72	− 2.10	− 1.18	0.60
** Max**	− 1.85	− 0.52	2.74	1.64	− 0.50	0.05	0.39	1.69
**Periodic mean concentrations**	− 2.56	− 0.85	1.86	0.61	− 0.89	− 0.85	− 0.52	1.15

#### The pollution load index

The *PLI* explained the overall pollution level of the pollutants of every sampling site in the study area (Cui et al., 2020). The highest HM *PLI* in the Manzala Lake sediments was reported in winter. The *PLI* values varied from 0.84 in site S7 to 2.26 in site S1 in winter, with an average value of 1.34. In summer, the values varied from 0.60 in site S7 to 1.69 in site S1, with an average value of 0.96 (Table 2, Fig. 3b). The periodic mean *PLI* value recorded in this study was greater than the threshold (< 1), indicating that the pollutants’ budgets were greater than the baseline values (Angulo, 1996). The maximum HM pollution loads were recorded following the location order S1 > S8 > S5 > S6 > S3 (values > 1) in winter and S1 > S8 (values > 1) in summer as a result of the diverse discharge of agricultural, industrial, and municipal wastes. Cd and Cu had high-risk concentration levels at every sampling site, which caused high distinct pollution *PLI* levels at specific sites.

#### The potential ecological (RI)

The potential ecological *RI* concerning the five HMs (Cd, Cu, Ni, Pb, and Zn) in the investigated area is shown in Fig. 3c, with values ranging from 115.9 at site S9 to 447.9 at site S1 with a mean value of 236.6 in winter. In summer, the RI values varied from 111.4 in site S9 to 338.5 in site S1, with a mean value of 174.9. These average values of *RI* in the sediments of the investigated area suggest a “moderate potential ecological risk.”

The average *Er* of the HMs followed a decreasing sequence: Cd > Cu > Pb > Ni > Zn. The values and toxicity of Cd alone at greater than 66% of the sampling locations in winter set a “high to very high potential ecological risk” status (Fig. 3c). Notably, Cd at site S1 was interpreted as a “very high potential ecological risk” in winter as the *Er* values reached 400 (> 320), suggesting great toxicity. This is in contrast with the four other metals, which were classified as “low potential ecological risks” (*Er* < 40) in winter and summer. The accumulation of these HMs can cause severe contamination in the bottom sediments of the lake. Despite the lowest concentrations of Cd compared with those of the other HM values in this study, the assessment outcomes gained from *I*_*geo*_ and *RI* suggest that Cd is a principal indicator of ecological risk. The coefficient of toxicity of Cd was 30 orders of magnitude greater than those of Zn and six orders of magnitude greater than those of Pb and Ni. Therefore, the contaminant level of a particular locality is controlled by the concentrations and toxicities of HMs.

### Possible sources

PCC is broadly applied to build assumptions regarding the probable sources of HMs in river and lake sediments (Zhang et al., 2016). The matrix correlations of the seven HMs and organic matter in the bottom sediments of the lake are shown in Tables 3 and 4. During winter, organic matter was significantly correlated (with very strong positive relationships) with Fe (*r* = 0.87, *p* < 0.01), Mn (*r* = 0.76, *p* < 0.05), Cd (*r* = 0.84, *p* < 0.01), Cu (*r* = 0.79, *p* < 0.05), Ni (*r* = 0.88, *p* < 0.01), Pb (*r* = 0.95, *p* < 0.01), and Zn (*r* = 0.82, *p* < 0.01). Meanwhile, Mn was moderately positively correlated with Cd (*r* = 0.66, *p* < 0.05). In summer, organic matter was significantly correlated (with very strong positive relationships) with Mn (*r* = 0.74, *p* < 0.05), Cd (*r* = 0.88, *p* < 0.01), Cu (*r* = 0.91, *p* < 0.01), Ni (*r* = 0.82, *p* < 0.01), Pb (*r* = 0.90, *p* < 0.01), and Zn (*r* = 0.90, *p* < 0.01) and moderately positively correlated with Fe (*r* = 0.61, *p* < 0.05). Meanwhile, Cd was moderately positively correlated with Mn (*r* = 0.52, *p* < 0.05) and Ni (*r* = 0.59, *p* < 0.05), and Fe was moderately positively correlated with Mn (*R* = 0.66, *p* < 0.05), Cd (*r* = 0.62, *p* < 0.05), Ni (*r* = 0.66, *p* < 0.05), and Pb (*r* = 0.53, *p* < 0.05). The coexistence of these metals with very strong positive linear relationships suggests identical geochemical natures or similar sources. The moderately positive correlation suggests a variable anthropogenic influence.

**Table 3:** PCC of HMs in winter and matrix of PC loadings after varimax rotation

**Variables**	**OM**	**Fe**	**Mn**	**Cd**	**Cu**	**Ni**	**Pb**	**Zn**	**PC1**	**PC2**
**OM**	1.00								**0.81**	− 0.48
**Fe**	0.87**	1.00							**0.71**	− 0.67
**Mn**	0.76**	0.87*	1.00						0.36	− **0.92**
**Cd**	0.84*	0.88*	0.66**	1.00					**0.90**	− 0.37
**Cu**	0.79*	0.91*	0.87*	0.89*	1.00				0.62	− **0.72**
**Ni**	0.88*	0.95*	0.87*	0.81*	0.82*	1.00			**0.67**	− 0.65
**Pb**	0.95*	0.93*	0.86*	0.85*	0.90*	0.89*	1.00		**0.72**	− 0.65
**Zn**	0.82*	0.92*	0.91*	0.81*	0.98*	0.83*	0.93*	1.00	0.56	− **0.80**
**Cumulative %**									88.33	93.16

The values in bold denote marked loading

*Significant at *p* < 0.01; **Significant at *p* < 0.05

**Table 4:** PCC of HMs in summer and matrix of PC loadings after varimax rotation

**Variable**	**OM**	**Fe**	**Mn**	**Cd**	**Cu**	**Ni**	**Pb**	**Zn**	**PC1**	**PC2**
**OM**	1.00								**0.82**	0.51
**Fe**	0.61	1.00							0.37	**0.72**
**Mn**	0.74**	0.66**	1.00						0.32	**0.91**
**Cd**	0.88*	0.62	0.52**	1.00					**0.91**	0.26
**Cu**	0.91*	0.71**	0.81*	0.82*	1.00				**0.76**	0.62
**Ni**	0.82*	0.66**	0.92*	0.59	0.82*	1.00			0.43	**0.85**
**Pb**	0.90*	0.53	0.66**	0.80*	0.94*	0.77*	1.00		**0.85**	0.43
**Zn**	0.90*	0.76**	0.80*	0.82*	0.99*	0.83*	0.92*	1.00	**0.75**	0.63
**Cumulative %**									80.82	89.34

The values in bold denote marked loading.

*Significant at *p* < 0.01; **Significant at *p* < 0.05

**Fig. 4:**
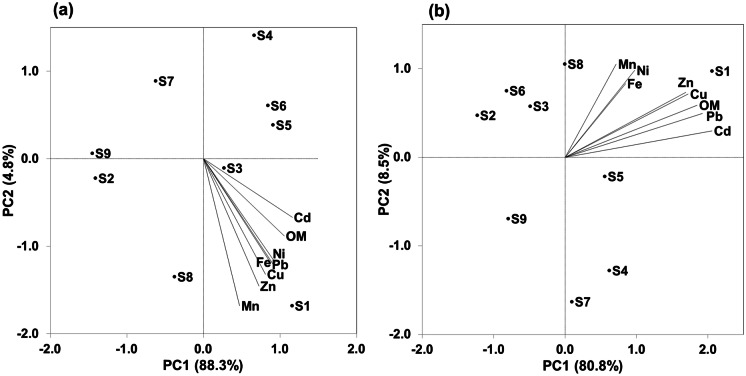
Loading plots of first two PCA of HMs in the sediments of Manzala Lake during the winter (**a**) and summer (**b**) seasons

PCA was utilized for the values of the HMs from the nine sites in Manzala Lake. The factors with cumulative contributions of > 80% of the variance were maintained, and high-loading HMs in every factor were decreased using varimax rotation to declare the probable sources. The outcomes are displayed in Tables 3 and 4 and Fig. 4. The first PCs 1 and 2 with eigenvalues > 1 were selected in winter and summer.

PC1 in winter had a principal rate of contribution, accounting for 88.3% of the whole variance, and was dominated by strong positive loading for Fe, Cd, Ni, Pb, and organic content, mostly at site S1. These indicate wastewater, agricultural, and/or industrial inputs (Belabed et al., 2013; El-Badry, 2016; Goher et al., 2017) (Table 3, Fig. 4a). PC2 in winter dominated by the strong negative loading of Mn, Cu, and Zn (accounting for 4.8% of the whole variance) was greatly linked at locations S2, S4, S7, and S9, indicating very low values for these HMs because of their confinement from urban inputs. In summer, PC1 was heavily loaded with Cd, Cu, Pb, Zn, and organic matter, accounting for 80.8% of the total variance, especially at location S1, indicating mostly wastewater and/or agricultural inputs (Belabed et al., 2013; El-Badry, 2016; Goher et al., 2017) (Table 4, Fig. 4b). Fe, Mn, and Ni (PC2, 8.5% of the whole variance) were greatly allied, exhibiting great positive loading at locations S2 (Al-Boghaz), S3 (Bashteer), S6 (El-Qapouti), and S8 (Al-Ginka). This probably relates to industrial activities and high rates of water inflow, mixing, metal remobilization at depth, and reprecipitation at the surface of these locations (Elkady et al., 2015; Wang et al., 2011) (Table 4, Fig. 4b). The combination of these HMs is predominantly anthropogenic in origin (Hu et al., 2013).

The distribution patterns of the HMs are not uniform because of the varied anthropogenic activities. The huge uncontrolled loads of untreated sewage, domestic, agricultural, and industrial wastes discharged into coastal lakes increase the concentration of most metals and lead to the continuous degradation of the water quality in these lakes (Abdel-Azeem et al., 2007; Abdel-Moati & El-Sammak, 1997; Elbehiry et al., 2018; Jaskuła & Sojka, 2022). These inputs can cause catastrophic impacts on the surrounding ecosystem as they pose dangerous health and environmental consequences and require proper management. Under the arid climate conditions in Egypt, low water flow from the AHD will not sluice the pollutants off of the Nile Delta again, therefore increasing the concentrations of heavy contaminants in Manzala and similar lakes. For instance, Cd and Pb disposed to the Nile Delta have expanded by 8–70 folds in the last 25 years (Abdel-Moati & El-Sammak, 1997). Moreover, pancreatic cancer risk in Manzala appears to be connected with high concentrations of Cd (Soliman et al., 2006). Great concentrations of Mn, Cu, Pb, and Zn can cause critical health concerns via the food chain, causing environmental and societal degradation (El-Rayis, 2005; Siegel, 1995).

## Conclusions

The concentrations, risk assessment, and probable origins of HMs in the bottom sediments of Manzala Lake, Egypt, were measured and assessed. The HM concentrations in the investigated area were exacerbated by different human impacts and activities. Organic matter and HM concentrations revealed abundance during the winter season. The average HM concentrations in the sediments conformed to the following sequence: Fe > Mn > Cu > Zn > Ni > Pb > Cd with great threat to the aquatic environment, especially due to Mn, Cd, Cu, and Ni enrichment. The geoaccumulation, pollution load, and ecological risk indices indicated pollution of the sediments with different HMs mainly during winter. Such human activities include agricultural, untreated municipal, and industrial waste discharge in huge quantities into the lake ecosystem increasing the concentration of most metals. PCA with cumulative contributions of > 80% of the variance declare the probable sources of HMs at the different lake stations. Site [S1] of Bahr Al-Baqar Drain is the highest contaminated site due to different wastewaters, agricultural, and/or industrial inputs. Manzala Lake is a highly polluted lake, and water liberated from the AHD will not sluice the pollutants off of the Nile Delta, therefore increasing the contamination level in the long term. The southern (Bahr Al-Baqar Drain, Bashteer, Legan, and Al-Ginka) and extreme northeastern (El-Qapouti) areas are heavily polluted by HMs compared with the northern parts.

Environmental pollution, degradation, and ecotoxicology of different metals, especially Cd, Cu, Mn, and Ni, affect the whole society in different ways. Thus, upgrading treatment technologies, prohibiting untreated wastes discharge, and continuous lake sediment monitoring are very important for future ecosystem management.

## Data Availability

The datasets generated during and/or analyzed during the current study are available from the corresponding author on reasonable request.
