# Correction to: Heavy metal pollution in Manzala Lake sediments, Egypt: sources, variability, and assessment

**DOI:** 10.1007/s10661-022-10129-1

**Published:** 2022-05-27

**Authors:** Mostafa Redwan, Engy Elhaddad

**Affiliations:** 1 Geology Dept, Faculty of Science, Sohag University, Sohag, 82524 Egypt; 2 National Institute of Oceanography and Fisheries, Cairo, Egypt

## Correction to: Environmental Monitoring and Assessment (2022) 194:1–16 https://doi.org/10.1007/s10661-022-10081-0

Upon publication of the original article, it was noticed that in the HTML version, the winter and summer symbols of Figs. 2 and 3 were the wrong way round.

The corrected Figs. 2 and 3 are shown in the next page.

**Fig. 2:**
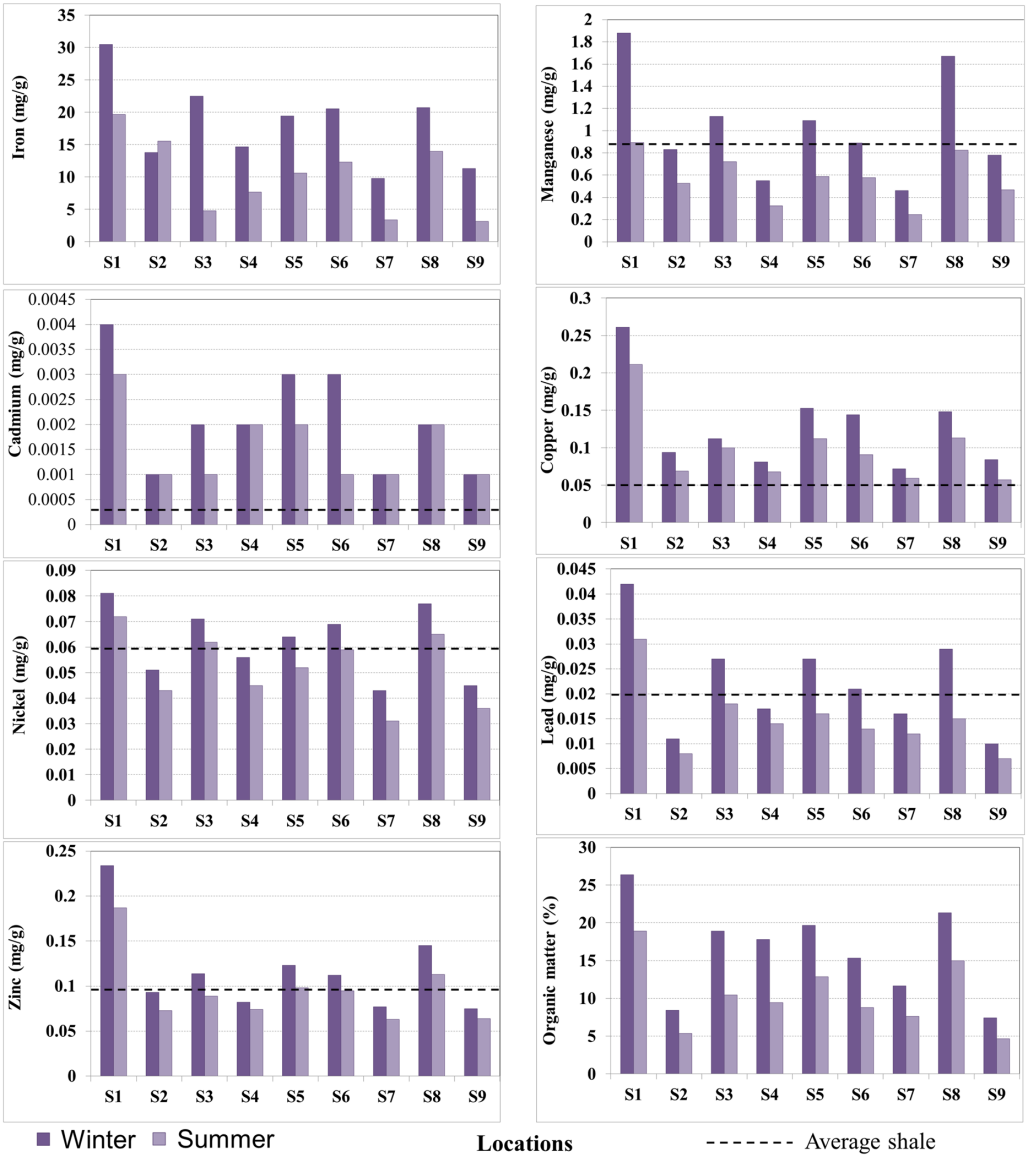
Total HM concentrations (mg/g) and organic matter (%) in sediments of Manzala Lake during winter and summer seasons. The dotted line represents average shale values (Turekian & Wedepohl, 1961)

**Fig. 3:**
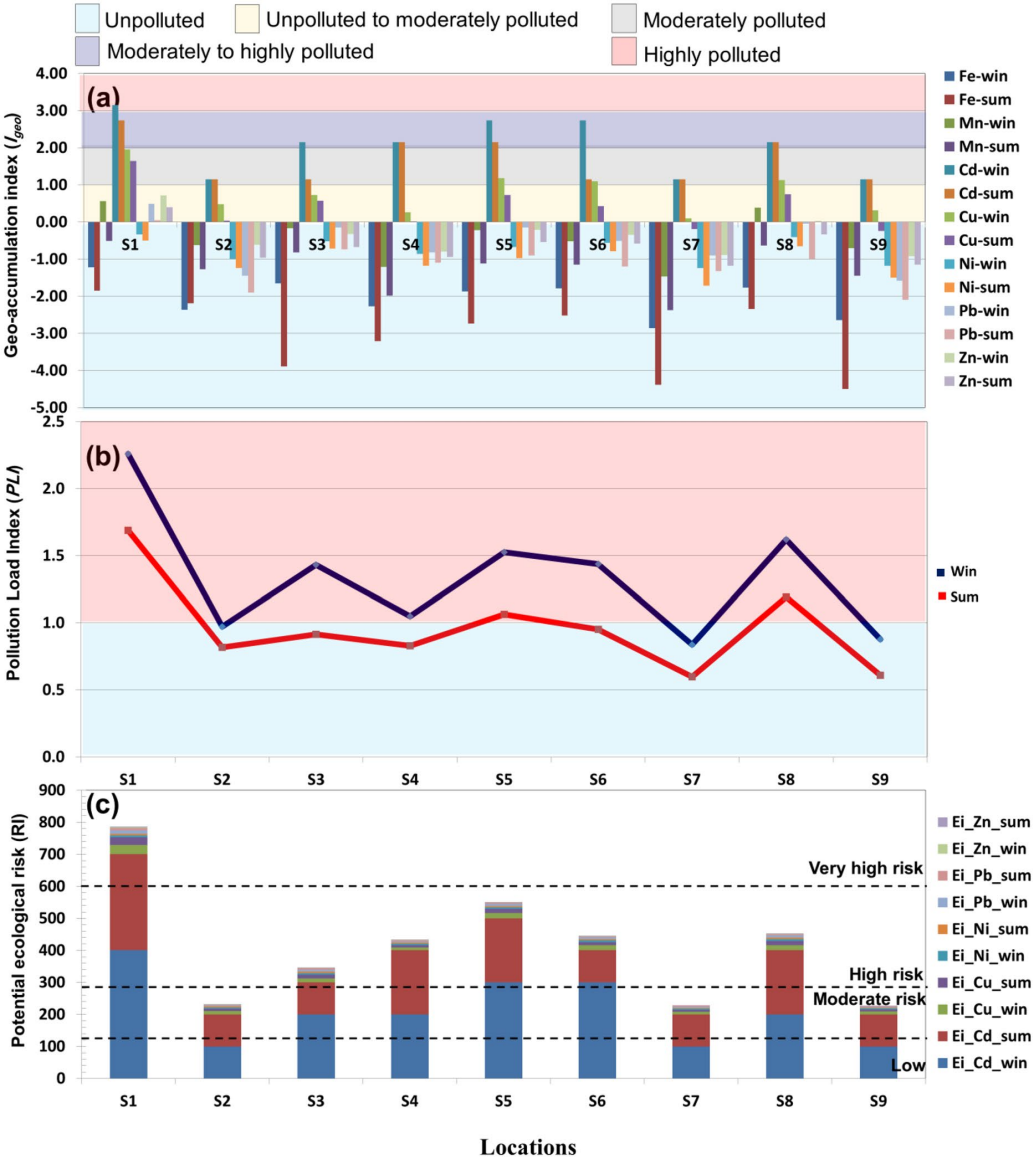
*I*_*geo*_ (**a**), *PLI* (**b**), and *RI* (**c**) of HMs in the sediments of Manzala Lake. Win, winter; Sum, summer

